# Recombinant adiponectin peptide promotes neuronal survival after intracerebral haemorrhage by suppressing mitochondrial and ATF4‐CHOP apoptosis pathways in diabetic mice via Smad3 signalling inhibition

**DOI:** 10.1111/cpr.12759

**Published:** 2020-01-10

**Authors:** Xun Wu, Jianing Luo, Haixiao Liu, Wenxing Cui, Wei Guo, Lei Zhao, Hao Guo, Hao Bai, Kang Guo, Dayun Feng, Yan Qu

**Affiliations:** ^1^ Department of Neurosurgery Tangdu Hospital The Fourth Military Medical University Xi'an China

**Keywords:** apoptosis, diabetes mellitus, intracerebral haemorrhage, recombinant adiponectin peptide, Smad3

## Abstract

**Objective:**

Low levels of adiponectin (APN), a biomarker of diabetes mellitus, have been implicated in the poor outcome of intracerebral haemorrhage (ICH). Herein, we aimed to demonstrate the neuroprotective effects of a blood‐brain barrier‐permeable APN peptide (APNp) on ICH injury in diabetic mice and explore the underlying mechanisms.

**Materials and methods:**

Recombinant APNp was administrated intraperitoneally to mice with collagenase‐induced ICH. Neurological deficits, brain water content and neural apoptosis were assessed. Western blotting, immunofluorescence staining, quantitative RT‐PCR and transmission electron microscopy were used to determine the signalling pathways affected by APNp.

**Results:**

Adiponectin peptide significantly alleviated neural apoptosis, neurological deficits and brain oedema following ICH in diabetic mice. Mechanistically, APNp promoted the restoration of peroxisome proliferator‐activated receptor gamma coactivator (PGC)‐1α related mitochondrial function and suppressed activating transcription factor 4 (ATF4)‐CCAAT‐enhancer‐binding protein homologous protein (CHOP)‐induced neural apoptosis. Furthermore, Smad3 signalling was found to play a regulatory role in this process by transcriptionally regulating the expression of PGC‐1α and ATF4. APNp significantly suppressed the elevated phosphorylation and nuclear translocation of Smad3 after ICH in diabetic mice, while the protective effects of APNp on mitochondrial and ATF4‐CHOP apoptosis pathways were counteracted when Smad3 was activated by exogenous transforming growth factor (TGF)‐β1 treatment.

**Conclusions:**

Our study provided the first evidence that APNp promoted neural survival following ICH injury in the diabetic setting and revealed a novel mechanism by which APNp suppressed mitochondrial and ATF4‐CHOP apoptosis pathways in a Smad3 dependent manner.

## 1 INTRODUCTION

Intracerebral haemorrhage (ICH) is an acute subtype of cerebral stroke with high death rates and severe neurological deficits. ICH‐induced poor outcomes result from complicated pathological processes that facilitate neural death. Diabetes mellitus (DM) is a proposed independent predictor of poor outcomes after ICH.^1^, ^2^ It is known to be connected with increased levels of neural apoptosis and also independently increased the risk of early death in patients with acute spontaneous ICH.^3^, ^4^, ^5^ Exploring the molecular basis that links diabetes with aggravated ICH injury and developing new therapeutic drugs to attenuate neural apoptosis are not only scientifically important but may also be of practical significance in the clinical setting.

Adiponectin (APN) is the main adipokine controlling the balance of energy metabolism at both cellular and systemic levels.^6^, ^7^, ^8^ Several studies have indicated that the concentration of plasma APN declines significantly in patients with type II diabetes and is also inversely correlated with poor outcomes of acute neural injury.^9^, ^10^, ^11^ However, till date, the protective effects of exogenous APN supplementation on aggravated ICH injury in a diabetic model remained unexplored. Due to the limited blood‐brain barrier (BBB) penetrability of the full‐length APN (a 30 kDa polypeptide), adenovirus‐mediated supplementation^12^, ^13^ and intracerebroventricular injection of APN^14^ were the primary methods adopted in previous studies; however, these methods of APN supplementation may not be suitable for clinical practice. In the present study, a variant APN peptide (APNp), which was synthesized in accordance with the amino acid sequence of the functional globular domain^15^, ^16^ at the C‐terminal end of APN amino acid sequence, was used.^17^, ^18^ Our previous studies proved that this peptide effectively crossed the BBB and functioned like endogenous APN after being intraperitoneally injected in mice.^17^

Mitochondrial dysfunction and endoplasmic reticulum (ER) stress have been acknowledged as the prominent features of several pathological conditions, including DM, obesity, fibrosis, neurodegenerative diseases, chronic inflammation, cancer and stroke.^19^, ^20^, ^21^ Mitochondrial dysfunction and ER stress induced by diabetes may render cells more susceptible to external stimuli and more prone to apoptosis.^22^, ^23^ Peroxisome proliferator‐activated receptor gamma coactivator (PGC)‐1α is a master regulator of mitochondrial functions and mitochondrial homoeostasis. It is critical for maintaining normal mitochondrial dynamics, mitochondrial oxidative respiration, energy metabolism and so on. Cytochrome c (Cyto.c) is an electron transport chain component that located in mitochondria. The release of Cyto.c from mitochondria into the cytoplasm is a vital step in mitochondrial apoptosis when the integrity of mitochondria was damaged.^24^ Impaired PGC‐1α function will facilitate the release of cytochrome c, which then contributes to mitochondrial apoptosis.^25^, ^26^ One vital step of ER stress‐mediated apoptotic pathway is the stimulation of pro‐apoptotic transcriptional factor C/EBP homologous protein (CHOP), also known as growth arrest and DNA damage‐inducible gene 153 (GADD153).^27^ CHOP, a transcriptional factor, has been demonstrated to regulate a great deal of pro‐ and anti‐apoptotic genes. One widely acknowledged mechanism of CHOP‐induced apoptosis is the decline of the pro‐survival protein Bcl2 and the elevation of pro‐apoptotic proteins Bax.^28^ Activating transcription factor 4 (ATF4) binds to the promoter of CHOP directly to activate its transcription29. Regulating ATF4‐CHOP axis induced apoptosis is of vital importance in numerous diseases.^29^, ^30^, ^31^ Moreover, the existence of correlations between mitochondrial and ER stress apoptosis pathways is generally accepted.^32^, ^33^ So, exploring the protective effects of APNp on mitochondrial and ATF4‐CHOP apoptosis pathways after ICH in db/db mice was one of the aims of our study.

Smad3 signalling has been indicated as a central mediator in numerous biological processes, including apoptosis, cell proliferation and energy metabolism. Considerable evidence demonstrates that the activation of Smad3 signalling correlates closely with the severity of metabolic diseases like obesity and diabetes in mice and humans.^31^, ^34^ However, the role played by Smad3 signalling in acute neural injury complicated with diabetes remained unclear. Smad3 is a transcription factor; once phosphorylated and activated, it translocates to the nucleus to bind to Smad‐binding elements (SBEs) in target gene promoters, influencing the transcription of the target genes and thus regulating various cellular processes.^35^, ^36^ Previous research used sequence analysis to reveal the presence of SBEs in the promoters of PGC‐1α and ATF4 and found that activated Smad3 signalling could transcriptionally decrease PGC‐1α levels^37^ and increase ATF4 levels^38^ in adipose tissue. This led us to question whether Smad3 played a distinctive role in regulating the mitochondrial and ATF4‐CHOP apoptosis pathways in diabetic mice that contribute to aggravated neural apoptosis following ICH.

Therefore, we aimed to investigate the neuroprotective effects of APNp treatment on neural survival following ICH injury in a mouse model of type 2 diabetes and to investigate the mechanisms by which APNp suppresses mitochondrial and ATF4‐CHOP apoptosis pathways in a Smad3‐dependent manner.

## 2 MATERIALS AND METHODS

### 2.1 Animals and ethical considerations

All experimental procedures were approved by Ethics Committee of Air Force Medical University and were conducted strictly in accordance to the guidelines of the National Institutes of Health Guide for the Care and Use of Laboratory Animals. Twelve weeks old, healthy, male C57BL/6J mice which weighed 20‐25 g were obtained from Animal Center of Air Force Medical University. Twelve weeks old, male db/db mice which weighed 40‐50 g were purchased from the Institute of Medical Biology, Chinese Academy of Medical Sciences. All mice were maintained in animal facilities under specific pathogen‐free conditions with 12 hours light/dark cycles, 18‐22°C, and given free access to food and water.

### 2.2 ICH surgery and drug administration

An in vivo collagenase‐induced ICH mouse model was used as described previously.^39^ Before ICH induction, mice were intraperitoneally injected with 15 mg/kg of APNp (Sangon Biotech Co.) dissolved in 0.9% saline solution twice (24 and 12 hours prior to ICH surgery) or with vehicle (equivalent dose of 0.9% saline) according to the groups assignment. Later, mice were anesthetized with 2% pentobarbital sodium and fixed using a mouse stereotaxic apparatus. 0.5‐mm‐diameter hole was drilled at the skull, and a solution of 0.075 U of collagenase VII‐S (Sigma‐Aldrich) in 0.25 μL saline was slowly injected into striata with stereotactic guidance (coordinates: 0.20 mm anterior, 2.30 mm right lateral, 3.50 mm deep) at a speed of 0.1 μL/min. Once this process was completed, the needle was left undisturbed for 5 minutes. The sham group was operated upon similarly but without collagenase injection. The exogenous transforming growth factor β1 (TGF‐β1) administration in vivo was performed as described previously^40^ with a modification. TGF‐β1 (7666 MB/CF, R&D Systems) was dissolved in PBS at 10 ng/μL, and each mouse was treated with 1 μL. TGF‐β1 was injected into striatum 10 minutes before ICH surgery at the same coordinates where ICH was induced.

### 2.3 Brain water content measurement

Mice brain water content was examined by the wet/dry method 24 hours after ICH.^41^ Brain water content was assessed 24 hours after ICH surgery as described previously. Briefly, after the mice were sacrificed, brains were removed and weighed to get the wet weight. Then brains were placed in an oven at 95‐100°C for 72 hours and weighed once more to get the dry weight. The percentage of brain water content was calculated as = (wet weight − dry weight)/wet weight × 100%.

### 2.4 Neurological outcomes

For comprehensive evaluation of neurological outcomes, three rating systems were used as described previously.^42^, ^43^ The modified neurological severity score was graded from score 0 to score 18 (normal mice: score 0; maximal deficit mice: score 18), with a higher score being indicative of a more remarkable neurological deficit. These scores were evaluated by two observers that were blind to the groups. The standard forelimb placing test was performed to measure the extent of motor and sensory impairment. Mice were tested 10 times for each forelimb, the percentage of experiments in which mice placed the appropriate forelimb on the edge of the countertop under vibrissae stimulation was recorded. Lastly, the corner turn test was performed, with each mouse being tested 10 times. Mice were allowed to proceed into 30° angled corner and recorded the percentage of right turns to get out of the 30° angled corner (only turns involving full rearing along each wall were contained).

### 2.5 Tissue preparation

Mice were sacrificed 24 hours after ICH induction. Following transcardial perfusion using 4% paraformaldehyde, mice brains were removed and dyed in 4% paraformaldehyde overnight at 4°C, after which 10%, 20% and 30% sucrose solutions were sequentially used for dehydration. Brain tissue was then sliced into 15‐ to 25‐μm sections for further experiments and analysis.

### 2.6 Fluoro‐Jade C (FJC) staining

Brain slices were prepared according to the procedures mentioned above. Selected slices were rehydrated in 80% ethanol containing 1% NaOH and 70% ethanol containing 1% NaOH for 5 minutes in turn, and washed with distilled water for 2 minutes. Slices were then dyed in 0.06% KMnO4 for 10 minutes and washed with distilled water for 3 minutes. Lastly, the slices were incubated in a 0.0001% solution of FJC (Millipore) for 15 minutes and washed with distilled water thrice (each time 1 minute). Images were captured with a fluorescence microscope (A1 Si, Nikon). FJC positive neurons around the haematoma were calculated by an independent observer that was blind to groups.

### 2.7 Terminal deoxynucleotidyl transferase‐mediated dUTP nick end‐labelling (TUNEL) staining

Brain slices were prepared according to the procedures mentioned above, and TUNEL staining for detecting cell apoptosis was performed in accordance with the manufacturer's protocols (Roche). Before the slices were incubated with TUNEL reaction mixture in the dark for 1 hour at 37°C, they were incubated at 37°C in 0.3% hydrogen peroxide for 0.5 hour and 0.25% proteinase K for 45 minutes, respectively. In the last step, slices were stained with 4,6‐diamidino‐2‐phenylindole (DAPI) (Invitrogen) solution for 10 minutes at 37°C. TUNEL‐positive neurons around the haematoma were counted by an independent observer who was blind to the experiment.

### 2.8 Immunofluorescence staining

Brain slices were prepared according to the procedures mentioned above. The selected brain slices were incubated in 0.1% Triton X‐100 for 0.5 hour and then blocked with PBS solution containing 5% goat serum (Gibco) for 30 minutes. The slices were then incubated at 4°C for 12 hours with primary antibodies—anti‐p‐Smad3 (1:200, ab52903, Abcam), anti‐NeuN (1:500, APN90P, Millipore). After being rinsed with PBS, the slices were incubated with corresponding secondary antibodies—donkey anti‐rabbit IgG (Alexa Fluor 488, Life Technologies), goat anti‐guinea pig IgG (Alexa Fluor 594, Life Technologies)—for 60 minutes at 25°C. Next, sections were stained using DAPI (Invitrogen) for 15 minutes at 25°C. In FITC‐labelled APNp (Sangon Biotech Co.) treated group, mice brain slices were directly incubated with DAPI for 15 minutes at 25°C. Finally, all sections were analysed with a fluorescence microscope (A1 Si, Nikon) by independent observers blinded to the experiment.

### 2.9 Transmission electron microscopy

Mice brain sample preparation was conducted as described previously.^44^ Briefly, mice were sacrificed after anaesthesia and successively perfused with 0.9% saline and 4% paraformaldehyde at 24 hours after ICH operation. The peripheral tissue of the cerebral haemorrhage was cut perpendicular to the long axis into blocks 1‐2 mm wide. Brain sections were dyed with 1% osmium tetroxide for 1 hour, soaked with graded ethanol for dehydration, post‐fixed with 4% glutaraldehyde overnight and implanted in resin. The tissue was then sliced into 80 nm brain sections with the ultramicrotome (Leica). Ultrathin sections were then fixed on 200‐slit grids that coated with pioloform membranes and observed with the JEM‐1400 electron microscope (JEOL); photomicrographs were obtained with the charge‐coupled device camera (Olympus).

### 2.10 Cell culture and treatments

Primary neurons were cultured from foetal C57BL/6 mice using the procedures described below.^37^ Corpus striata were separated and digested with 0.125% trypsin for half an hour, and then 500 μL of this suspension was added into a 24‐well plate which was pre‐coated with poly‐L‐lysine. Cells were cultured in Dulbecco's modified Eagle's medium containing 1% L‐glutamate, 10% foetal bovine serum (FBS) and 1% penicillin‐streptomycin in 5% CO2 for 4 hours. The supernatant was then replaced with 500 μL Neurobasal medium containing 1% penicillin‐streptomycin, 1% L‐glutamate, 2% B27 and 1% N2 for 12 days. Change half of the medium every other day. Cells were serum starved for 24 hours and then incubated with high glucose (HG) (100 mmol/L) medium without FBS for 24 hours to stimulate in vitro hyperglycaemia. Next, the cells were treated with 50 μmol/L APNp or 5 μg/mL TGF‐β1 for 12 hours before treatment with 20 μmol/L oxyhaemoglobin (OxyHb) for 6 hours.

### 2.11 Quantitative real‐time PCR (qRT‐PCR)

Extracted Total RNA with TRIzol Reagent (Invitrogen) in accordance to the manufacturer's instructions. Hiscript II Q RT SuperMix for qRT‐PCR (+gDNA wiper) (Vazyme) was used to reverse transcribe the RNA (1 mg). qRT‐PCR was performed with the iQTM 5 Optical Module Real‐Time PCR Detection System (Bio‐Rad). mRNA was quantified using the ChamQtm SYBR qPCR master mix according to the manufacturer's instructions. The $2^{-\Delta \Delta C_{t}}$ method was used to quantitate the relative gene expression changes normalized to β‐actin.

### 2.12 Mitochondrial functional analysis

Measurement of mitochondrial membrane potential via tetramethyl rhodamine ethyl ester (TMREM) staining and measurement of mitochondrial ROS generation via MitoSox Deep Red staining was performed as described previously.^45^ Five nanomolar MitoSox (M36008, Invitrogen) or 10 nmol/L TMRE (T669, Life Technologies) was used to incubate with primary neuron for 30 minutes. After that, cells were washed three times with HBSS to remove the excess dye. Then, a fluorescence microscope (A1 Si, Nikon) was used to capture pictures and ImageJ software was used to quantify the relative fluorescence levels.

### 2.13 Western blot

The selected samples were collected and homogenized in lysis buffer containing 1% protease inhibitor. Protein concentrations were measured using a BCA Protein Assay kit (Thermo Scientific). Protein samples were separated on sodium dodecyl sulphate‐polyacrylamide gel electrophoresis gels and transferred to polyvinylidene fluoride membranes (Millipore). Following blocking in 5% skim milk solution in tris‐buffered saline with Tween 20 (TBST), incubated the membranes with primary antibody at 4°C for 12 hours. Next, incubated the membranes with the corresponding horseradish peroxidase‐conjugated secondary antibodies (1:5000, WH112425, ABclonal) for 2 hours, followed by three 5 minutes TBST washes. Protein bands were visualized using the BioRad imaging system (Bio‐Rad). The following primary antibodies were used: anti‐p‐Smad3 (1:1000, ab52903, Abcam), anti‐Smad3 (1:1000, ab28379, Abcam), anti‐ATF4 (1:1000, D4B8, Cell Signaling), anti‐CHOP (1:1000, L63F7, Cell Signaling), anti‐VDAC (1:1000, D73D12, Cell Signaling), anti‐PGC‐1α (1:1000, 4C1.3, Calbiochem), anti‐Bax (1:1000, gtx32465, Gene Tex), anti‐Bcl2 (1:1000, gtx100064, Gene Tex), anti‐cytochrome c (1:1000, wh118104, Wanleibio), anti‐β‐actin (1:3000, wh096194, Wanleibio), anti‐GAPDH (1:3000, LM16989, Proteintech).

### 2.14 Statistical analysis

Kruskal‐Wallis one‐way analysis of variance (ANOVA) on ranks followed by the Student‐Newman‐Keuls test was used for neurobehavioral data analysis. Student's *t* test (unpaired, two‐tailed) was used to analyse the statistical differences between two groups. Comparison among multiple groups was analysed using ANOVA followed by the Tukey post hoc test. *P*‐values <.05 were considered statistically significant. All data are expressed as mean ± standard deviation. SPSS (version 21.0) was used to analyse the data.

## 3 RESULTS

### 3.1 APNp treatment attenuated neurological function deficits and relieved brain oedema after ICH in db/db mice

**Figure 1:**
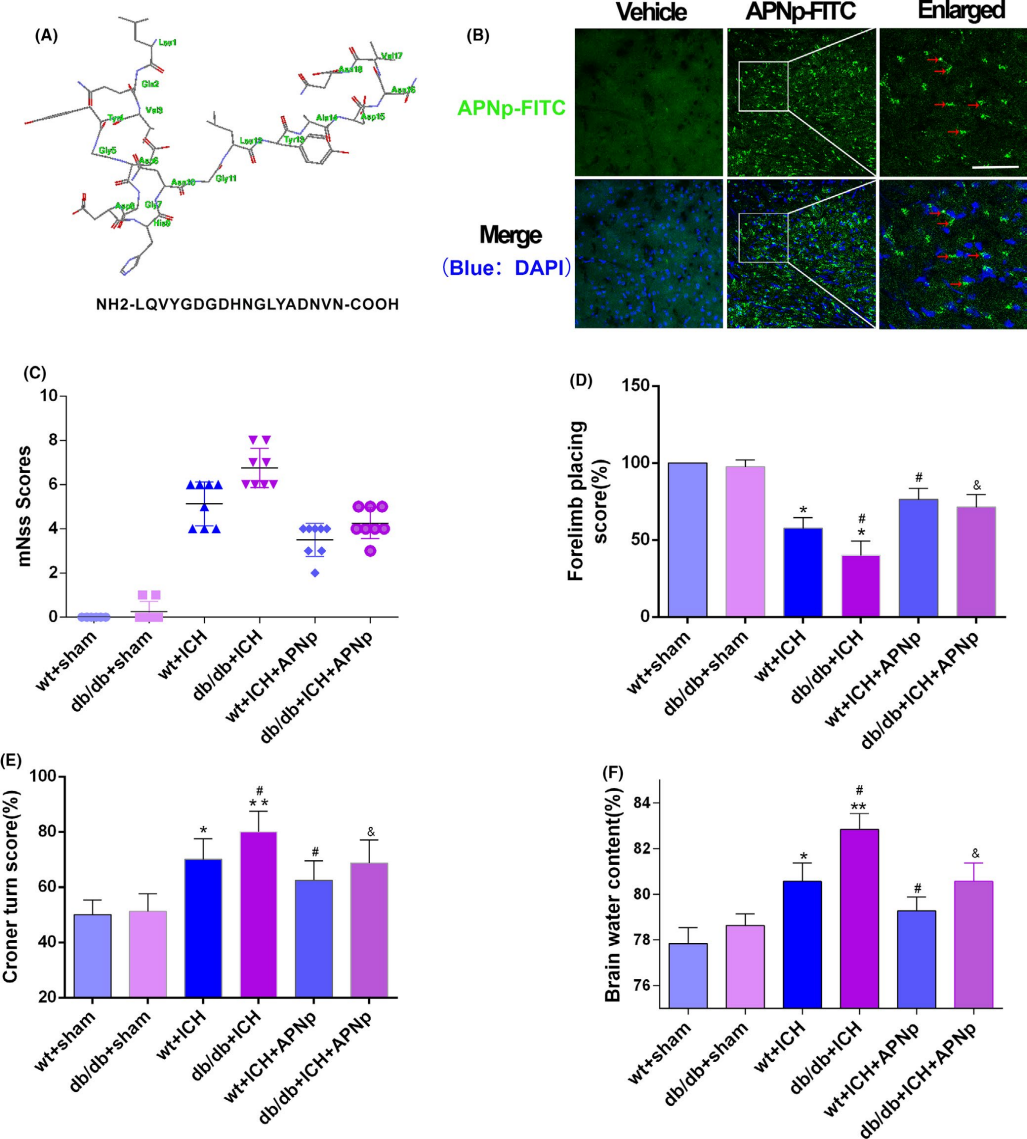
Diabetes aggravated neurological function deficits and brain oedema after intracerebral haemorrhage (ICH), which were significantly reversed by adiponectin peptide (APNp) treatment. A, The amino acid sequence of APNp. B, Immunofluorescence image of fluorescein isothiocyanate (FITC)‐labelled APNp in db/db mice brain 24 h after intraperitoneal administration. Blue: DAPI. Red arrows indicated (FITC)‐labelled APNp. Scale bar: 100 μm. The boxed area besides each micrograph represents the amplification of the region within the white square. C, Modified neurological severity scores. D, Forelimb placing test results. E, Corner turn test results. F, Brain water content measured at 24 h after ICH. Values were represented as mean ± SD, n = 8 for each group. *P < .05 and **P < .01 vs wt+sham group, ^#^ *P* < .05 vs wt+ICH group, ^&^ *P* < .05 vs db/db+ICH group

The amino acid sequence of APNp is shown in (Figure 1A). FITC‐labelled APNp was intraperitoneally injected in db/db mice, and immunofluorescence analysis was conducted 24 hours later to verify the effective BBB penetration of APNp (Figure 1B); neurological function and brain water content were also assessed. Both nondiabetic mice and db/db mice showed significant neurological function deficits after ICH; however, the sensorimotor function deficits in db/db ICH mice were significantly worse than those in nondiabetic ICH mice. Importantly, APNp treatment dramatically attenuated deficits in both nondiabetic ICH mice and db/db ICH mice, and the improvement in db/db mice was more significant than that in nondiabetic mice (Figure 1C‐E). We found that db/db ICH mice had a significantly higher brain water content (5.9% increase vs wt+sham) than nondiabetic ICH mice (3.1% increase vs wt+sham). Interestingly, APNp significantly reduced brain oedema in both nondiabetic mice (1.1% reduction vs wt+ICH) and db/db mice (2.5% reduction vs db/db+ICH) (Figure 1F).

### 3.2 APNp treatment protected the ultrastructure of neurons and promoted neural survival after ICH in db/db mice

**Figure 2:**
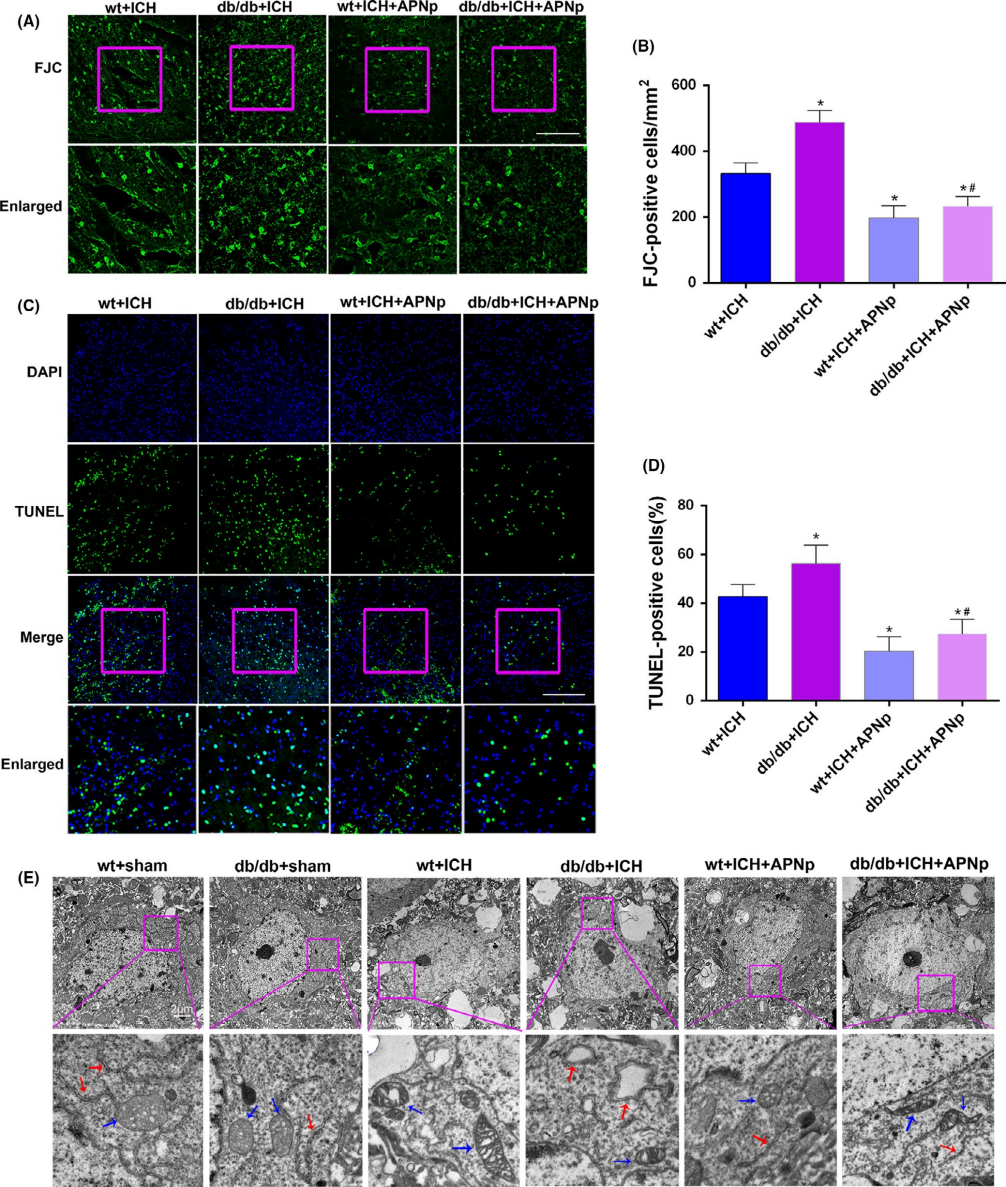
Diabetes aggravated neuronal damage after ICH, which was significantly reversed by APNp treatment. A, Representative images of Fluoro‐Jade C (FJC) staining and B, quantitative analyses of FJC positive neurons 24 h after ICH induction. Scale bar: 100 μm. C, Representative images of terminal deoxynucleotidyl transferase dUTP nick end‐labelling (TUNEL) staining and D, quantitative analyses of the percentage of TUNEL‐positive cells 24 h after ICH induction. Scale bar: 200 μm. E, Electron microscopy was performed to observe ultrastructural changes of neurons in mice. The boxed area under each micrograph represents the amplification of the region within the pink square. Blue arrows represent mitochondria, and red arrows represent the endoplasmic reticulum (ER). Values are represented as mean ± SD. n = 8 for each group. *P < .05 vs wt+ICH group, ^#^ *P* < .05 vs db/db+ICH group

The degenerated neurons were quantified by counting the number of FJC‐positive cells. Both nondiabetic mice and db/db mice exhibited an increase in the number of FJC‐positive neurons after ICH. Moreover, the increase in db/db ICH mice was more significant than that in nondiabetic ICH mice. Importantly, APNp treatment significantly reduced the ICH‐induced elevated neuronal degeneration both in nondiabetic and diabetic mice by a percentage of 40.7% and 52.4%, respectively (Figure 2A,B, *P* < .05). In addition, we found that db/db ICH mice exhibited more neural apoptosis than the nondiabetic ICH mice. APNp treatment dramatically attenuated the percentage of TUNEL‐positive neurons in both nondiabetic mice (22.3% reduction) and db/db mice (29.0% reduction) (Figure 2C,D, *P* < .05). Transmission electron micrographs were obtained for studying the ultrastructural changes of neurons in each group. Our results show that the neurons were characterized by mitochondrial cristae loss, swollen mitochondria and morphological alterations in the ER after ICH. Furthermore, APNp treatment significantly protected against ICH‐induced morphological damage both in nondiabetic mice and db/db mice (Figure 2E). These data indicated that diabetes exacerbated neuronal degeneration, apoptosis and morphological destruction after ICH, which could be reversed significantly by APNp treatment.

### 3.3 APNp promoted the restoration of PGC‐1α related mitochondrial function and suppressed the ATF4‐CHOP axis after ICH in db/db mice

**Figure 3:**
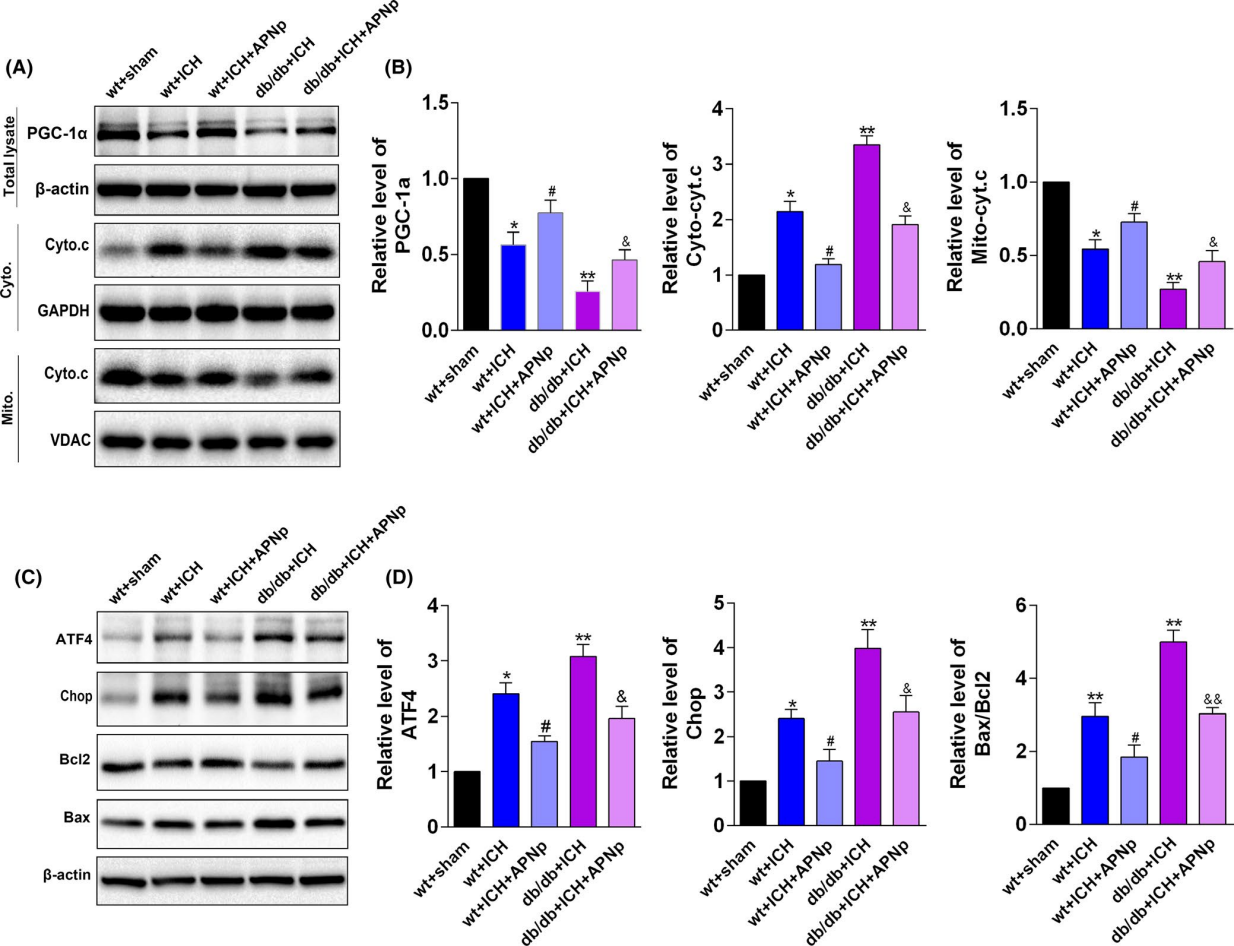
Diabetes aggravated mitochondrial dysfunction and ER stress after ICH, which were significantly reversed by APNp treatment. A, B, Western blotting analysis of the level of peroxisome proliferator‐activated receptor gamma coactivator (PGC)‐1α, mitochondrial cytochrome c and cytoplasmic cytochrome c. C, D, Western blotting analysis of the levels of activating transcription factor 4 (ATF4), CCAAT‐enhancer‐binding protein homologous protein (CHOP), Bcl2‐associated X (Bax) and B‐cell lymphoma 2 (Bcl2). Values are represented as mean ± SD, n = 8 for each group. *P < .05 and **P < .01 vs wt+sham group, ^#^ *P* < .05 vs wt+ICH group, ^&^ *P* < .05 and ^&&^ *P* < .01 vs db/db+ICH group

The protein levels of PGC‐1α, a regulator of mitochondrial biogenesis, were decreased after ICH in both nondiabetic mice and db/db mice, and the decline in db/db ICH mice was more significant than that in the nondiabetic ICH mice (Figure 3A,B, *P* < .05). Consistent with this, ICH significantly facilitated the release of cytochrome c from mitochondria to cytoplasm, a vital step in the mitochondrial apoptosis pathway, in both nondiabetic mice and db/db mice (Figure 3A,B, *P* < .05). Additionally, the ER was also monitored. Consistent with the inhibition of the ATF4‐CHOP axis, the downstream anti‐apoptosis protein Bcl2 was down‐regulated, and the pro‐apoptosis protein Bax was up‐regulated (Figure 3C,D, *P* < .05). Importantly, APNp significantly protected against mitochondrial dysfunction and ER stress after ICH injury in diabetic mice, as was evidenced by the improved PGC‐1α level, decreased release of cytochrome c, suppressed ATF4‐CHOP axis and the normalization of Bax/Bcl2 (Figure 3A‐D, *P* < .05).

### 3.4 APNp treatment suppressed the elevated phosphorylation and nuclear translocation of Smad3 after ICH in db/db mice

**Figure 4:**
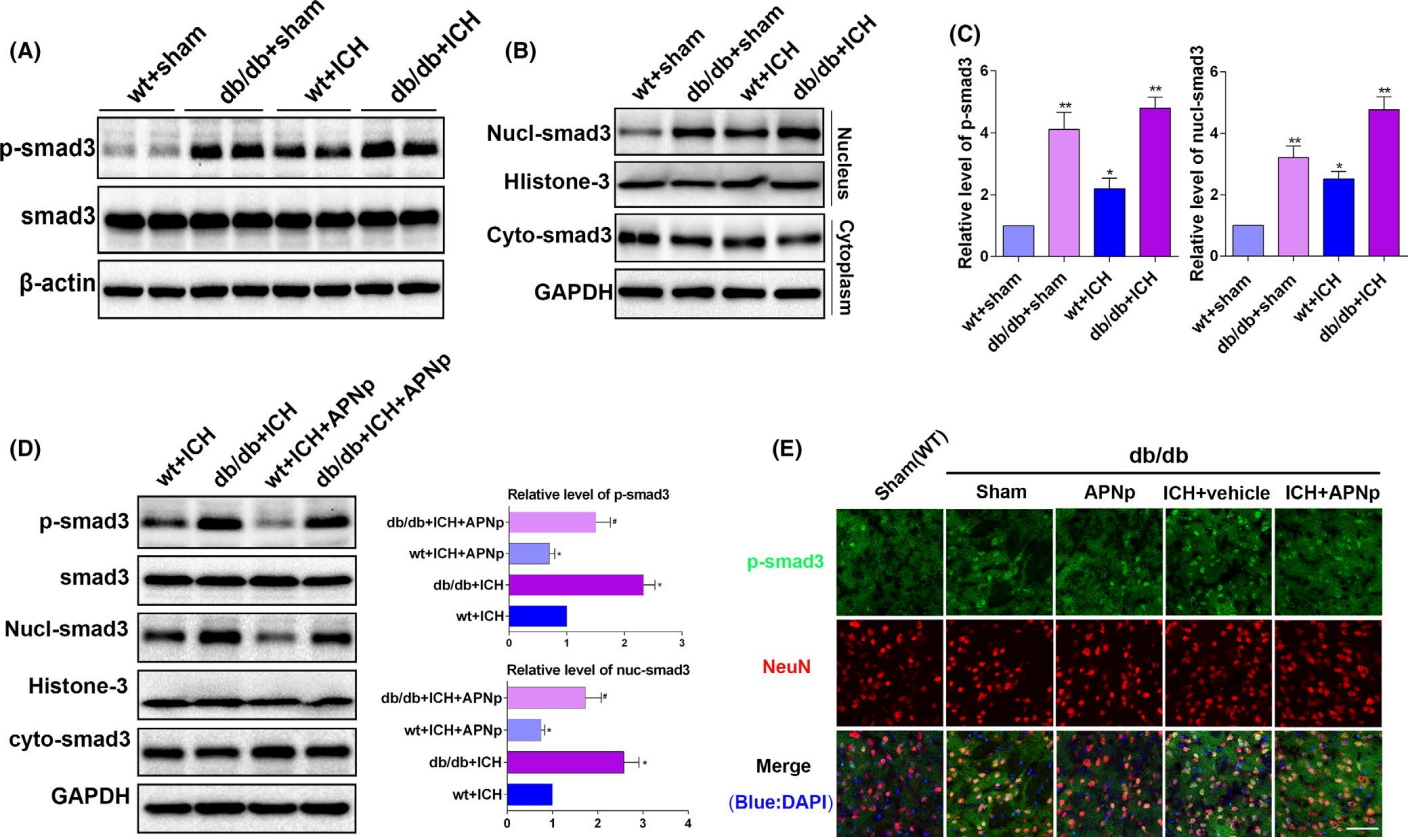
Diabetes aggravated the phosphorylation of Smad3 after ICH, which was significantly reversed by APNp treatment. A, C, Western blotting analysis of Smad3 phosphorylation level. B, C, After the cyto‐nuclear separation, Western blotting was used to analyse the nuclear translocation of Smad3. D, Western blotting analysis of the effects of additional APNp treatment on the phosphorylation level and the nuclear translocation of Smad3 after ICH. E, Representative images of double immunofluorescence staining for p‐smad3 (green) with NeuN (red) in the perihaematomal area 24 h after ICH are shown. Scale bar: 100 μm. Values are represented as mean ± SD, n = 8 for each group. *P < .05 and **P < .01 vs wt+sham group, *^#^P* < .05 vs wt+ICH group

Western blot indicated that Smad3 was robustly phosphorylated in db/db mice, and phosphorylation was further enhanced after ICH (Figure 4A,C, *P* < .01), while the baseline Smad3 level remained unchanged. Notably, the elevated phosphorylated (p)‐Smad3 level in db/db ICH mice was more significant than that in the nondiabetic ICH mice, indicating that Smad3 signalling may play a distinctive role in ICH injury with diabetes. After the cyto‐nuclear separation, the increased nuclear translocation of Smad3 in db/db mice was confirmed (Figure 4B,C, *P* < .05), which was consistent with the elevation of p‐Smad3. Importantly, APNp efficiently inhibited the enhanced phosphorylation of Smad3 and suppressed its nuclear translocation after ICH insults both in nondiabetic mice and in db/db mice (Figure 4D, *P* < .05), and the results of immunofluorescence analysis were consistent with those of Western blotting (Figure 4E). These data revealed that Smad3 signalling might play a distinctive role in the pathological process of ICH injury with diabetes and that its activity was subject to the regulation of APN.

### 3.5 Elevated Smad3 signalling aggravated mitochondrial dysfunction and ER stress under HG and OxyHb stimulation through transcriptionally targeting PGC‐1α and ATF4, respectively

**Figure 5:**
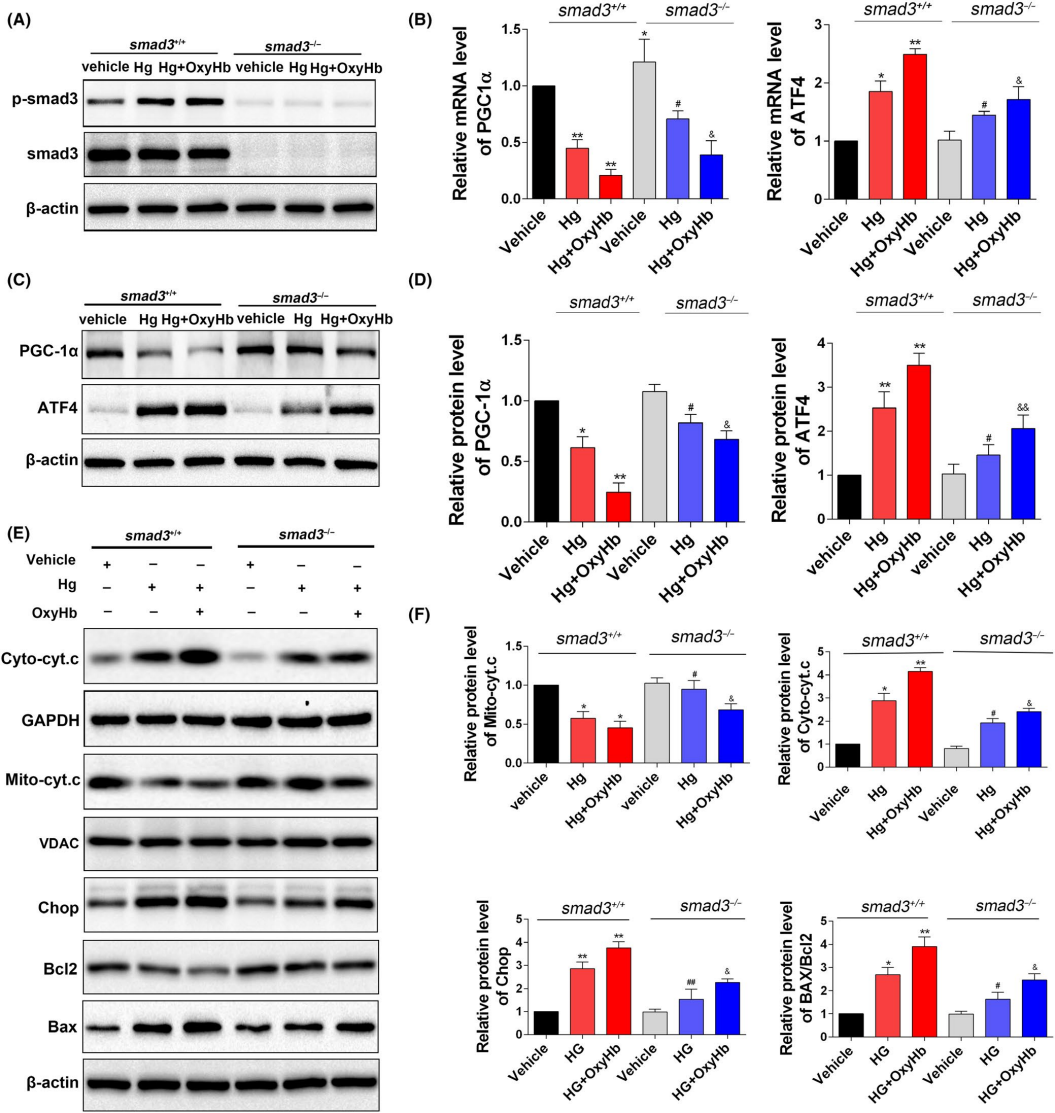
Smad3 knockout alleviated mitochondrial dysfunction and ER stress induced by high glucose and oxyhaemoglobin (OxyHb) stimulation through transcriptionally targeting PGC‐1α and ATF4, respectively. The *Smad3* knockout 293T cell line was used for studying the underlying molecular mechanisms. A, Western blotting analysis of p‐Smad3 and Smad3. B, mRNA levels of PGC‐1α and ATF4. C, D, Western blotting analysis of the protein levels of PGC‐1α and ATF4. E, F, Western blotting analysis of the levels of mitochondrial cytochrome c, cytoplasmic cytochrome c, CHOP, Bax and Bcl2. Values are represented as mean ± SD, n = 8 for each group. *P < .05 and **P < .01 vs vehicle (*Smad3* ^+/+^) group, ^#^ *P* < .05 and ^##^ *P* <.01 vs Hg (*Smad3* ^+/+^) group, ^&^ *P* < .05 and ^&&^ *P* < .01 vs Hg+OxyHb (*Smad3* ^+/+^) group

The *Smad3* knockout 293T cell line was used for further study of the underlying molecular mechanisms. In wild type 293T cells, Smad3 was significantly phosphorylated in the HG state (Figure 5A, *P* < .05 vs the vehicle group), and this was further enhanced after OxyHb treatment (Figure 5A, *P* < .01). Previous studies indicated that Smad3 could transcriptionally regulate PGC‐1α and ATF4 by binding to their gene promoters. In the current study, consistent with the elevated p‐Smad3, we found that both the protein level and mRNA level of PGC‐1α were significantly decreased under HG condition and OxyHb treatment (Figure 5B‐D, *P* < .05); conversely, in the Smad3 knockout group, the decline was significantly alleviated (Figure 5B‐D, *P* < .05). Smad3 knockout group also exhibited reduced cytochrome c release (Figure 5E,F, *P* < .05). Moreover, Smad3 knockout alleviated both the elevated mRNA and protein levels of ATF4 induced by HG and OxyHb stimulation (Figure 5B‐D, *P* < .05). Consistent with this, Smad3 knockout inhibited the activation of the CHOP pathway, reversed the decline of its downstream target Bcl2, and suppressed the elevation of Bax (Figure 5E,F, *P* < .05). These results revealed that Smad3 signalling may be essential for the initiation of mitochondrial dysfunction and ER stress under HG and OxyHb stimulation via transcriptionally decreasing PGC‐1α and increasing ATF4, respectively.

### 3.6 The protection of APNp on mitochondrial and ATF4‐CHOP apoptosis pathways was neutralized when Smad3 was activated by additional transforming growth factor (TGF)‐β1 treatment both in vitro and in vitro

**Figure 6:**
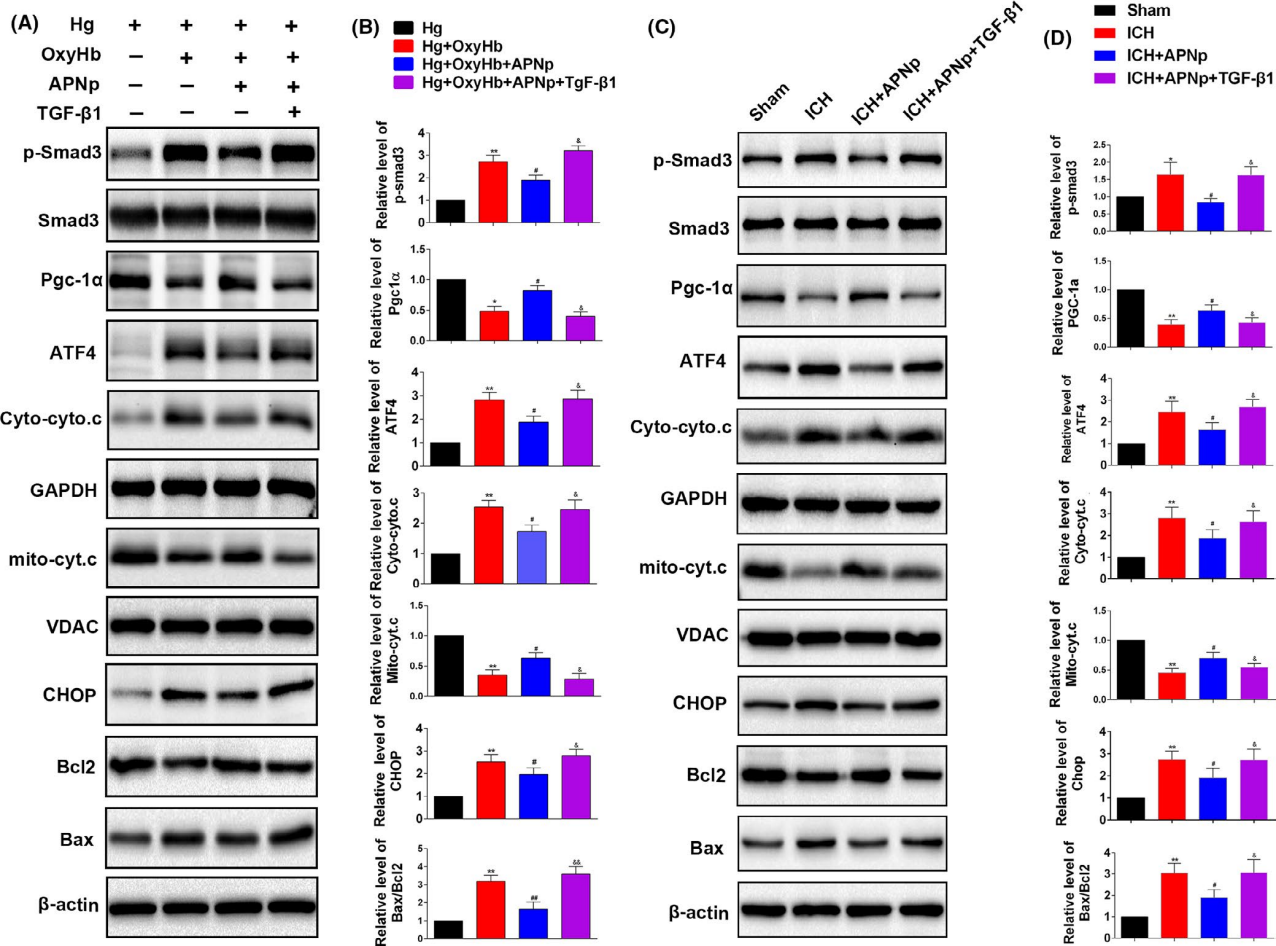
The protection of APNp on mitochondrial function and ER stress was neutralized when Smad3 was activated by additional transforming growth factor (TGF)‐β1 treatment both in vitro and in vivo. A, B, Western blotting analysis level of p‐Smad3, Smad3, PGC‐1α, ATF4, Mito‐Cyto.c, Cyto‐Cyto.c, CHOP, Bax and Bcl2 of each group in primary neuron. C, D, Western blotting analysis level of p‐Smad3, Smad3, PGC‐1α, ATF4, Mito‐Cyto.c, Cyto‐Cyto.c, CHOP, Bax and Bcl2 of each group in diabetic mice. Values are represented as mean ± SD, n = 8 for each group. *P < .05 and **P < .01 vs Hg (high glucose) +vehicle group, ^#^ *P* < .05 and ^##^ *P* < .01 vs Hg+OxyHb group, ^&^ *P* < .05 and ^&&^ *P* < .01 vs Hg+OxyHb+APNp group

We next explored whether Smad3 signalling was indispensable for APNp to protect against the mitochondrial and ATF4‐CHOP apoptosis pathways. Primary neurons were incubated with an increased concentration of glucose (100 mmol/L) for 48 hours to simulate an in vitro hyperglycaemic model, and then other treatments followed. Firstly, we found that APNp can significantly suppress the elevated level of p‐Smad3 after additional OxyHb treatment (Figure 6A,B, *P* < .05). Meanwhile, the protection of additional APNp treatment on PGC‐1α related mitochondrial function and ATF4‐CHOP axis (Figure 6A,B, *P* < .05) after additional OxyHb stimulus were identified. Secondly, additional TGF‐β1 treatment, which significantly stimulated the activities of Smad3, counteracted the protection of APNp on PGC‐1α expression and Cyto.c release. Meanwhile, the protection of APNp on the ATF4‐CHOP axis and its downstream proteins Bcl2 and Bax were also neutralized following additional TGF‐β1 stimulation (Figure 6A,B, *P* < .05). Correspondingly, in diabetic mice, additional TGF‐β1 treatment also neutralized the protection of APNp on PGC‐1α expression, Cyto.c release, ATF4‐CHOP axis and its downstream proteins Bcl2 and Bax following ICH. (Figure 6C,D, *P* < .05).

## 4 DISCUSSION

The complexity and heterogeneity of ICH, as well as of the associated comorbidities, may render neuroprotective drugs less efficient in clinical practice. Therefore, developing targeted therapeutic strategies to promote neural cell survival and improve neurological function are of great significance to the outcome of ICH. The major findings of the present experiments can be summarized as follows: (a) compared with nondiabetic mice, db/db mice presented some confounding pathological features of ICH, including more severe brain oedema and neurological deficits, aggravated mitochondrial and ATF4‐CHOP apoptosis injuries, and exacerbated activation of Smad3 signalling. (b) APNp treatment might be a novel therapeutic strategy for brain injury (as was evidenced by reduced brain oedema and improved neurological function) following ICH complicated with type 2 diabetes. (c) APNp treatment remarkably alleviated mitochondrial dysfunction and ATF4‐CHOP axis induced apoptosis after ICH in diabetic mice, and (d) mechanistically, this alleviation was in a Smad3‐dependent manner.

Besides its acknowledged function in the maintenance of metabolic homoeostasis, APN also possesses anti‐inflammatory, antioxidant and antiatherogenic properties.^46^, ^47^ Reduced APN function has been demonstrated to be a major contributor in the pathogenic process of type 2 DM; meanwhile, clinical trials showed that the level of serum APN was negatively correlated with acute death and functional impairment after cerebral stroke.^9^, ^10^, ^11^ All these observations indicated that reduced APN function might play a causative role in aggravated brain damage in diabetic patients after ICH, this inspired us to hypothesize that exogenous supplementation of APN could act as a promising therapeutic strategy. Considering the restricted BBB permeability of full‐length APN and the feasibility for future clinical applications, a recombinant APNp was synthetized and used in the present study. We found that APNp significantly improved the neurological function, attenuated brain oedema and promoted neuronal survival, indicating the promising neuroprotective effects of APNp on ICH injury in patients with diabetes.

Excessive neural apoptosis is an important cause of neurological function deficits and poor outcome after ICH. The appearance of mitochondrial apoptosis and ER stress‐induced apoptosis has been considered as one of the prominent features of both metabolic disorders and acute neural injury. The failure of PGC‐1α function, a regulator of mitochondrial biogenesis, invariably leads to mitochondrial apoptosis.^25^ And the ATF4‐CHOP axis is demonstrated to be a main pathway contributing to ER stress induced apoptosis.^29^ In this study, we found that compared with the nondiabetic mice, mitochondrial dysfunction and ER stress induced apoptosis after ICH were more profound in diabetic mice. This may, at least partly, account for the fact that the recovery of ICH patients with diabetes is often less satisfactory when compared with that of patients in the normal population. Notably, we also found that APNp treatment could maintain mitochondrial stability and alleviate ER stress, as was evidenced by improved PGC‐1α function and suppressed ATF4‐CHOP signalling. These results support the hypothesis that exogenous supplementation of APN to the brain is a promising therapeutic strategy to promote neural survival.

We also went further to investigate which possible key regulator of both mitochondrial and ER stress apoptosis pathways could be regulated by APNp for its anti‐apoptosis effects. Smad3 signalling plays a critical role in the pathological process of diabetic diseases; in the current study, we found that Smad3 was robustly phosphorylated in the brain of db/db mice and that phosphorylation was further enhanced after ICH. This led us to the hypothesis that Smad3 activation may play a distinctive role in aggravated ICH injury in diabetes. Previous research indicated that activated Smad3 could transcriptionally suppress PGC‐1α and upregulate ATF4 in adipose tissue,^37^, ^38^ impairing mitochondrial function and inducing ER stress related apoptosis. In the current study, we demonstrated that Smad3 knockout significantly alleviated HG and OxyHb induced mitochondrial dysfunction and ATF4‐CHOP axis signalling to a large extent in in vitro experiments. As Smad3 may function as a critical regulator of mitochondrial and ER stress apoptosis pathways in the diabetic setting, we next explored whether the protection of APNp on mitochondrial dysfunction and ER stress depended on the inhibition of Smad3 signalling. Both in vivo and in vitro, we found that APNp could significantly suppress the elevated Smad3 phosphorylation, which is consistent with a previous study that APN inhibited phosphorylation of Smad3 in mesangial cells.^48^ TGF‐β1 treatment to stimulate Smad3 activity could neutralize APNp protection on mitochondrial dysfunction and ER stress. This further demonstrated that APNp protected mitochondrial dysfunction and ER stress by inhibiting Smad3 signalling in the diabetic setting.

One purpose of this study was to clarify the distinction between two different conditions and identify the detailed molecular mechanism that links diabetes with aggravated ICH. Although neural apoptosis, mitochondrial dysfunction and ER stress are also secondary to ICH without nondiabetic mice, these events seemed to be more remarkable in diabetic mice (Figures 2 and 3). And in the study, we identified Smad3 signalling as a potential vital factor that links diabetes with aggravated ICH. Specifically, aggravated Smad3 signalling dependent mitochondrial and ATF4‐CHOP apoptosis pathways may be the underlying mechanism that link diabetes with aggravated ICH. This finding may not only scientifically important but may also be of guiding significance in future clinical treatment.

Notably, numerous researches indicated the correlation between mitochondrial and ER stress apoptosis pathways.^32^, ^33^ Will there exist a crosstalk between the mitochondrial and ATF4‐CHOP apoptosis pathways worth our exploration. The activation of ATF4‐CHOP could induce the down‐regulation of Bcl2 and the up‐regulation of Bax which also play a critical role in mitochondrial apoptosis. Specifically, one consequence of the down‐regulation of Bcl2 and the up‐regulation of Bax is the mitochondrial outer membrane permeabilization (MOMP), the impairment of mitochondrial integrity and the release of apoptogenic proteins into the cytoplasm, such as cytochrome c and SMAC. These process induced apoptosome formation and caspase activation, thus leading to mitochondrial apoptosis.^49^, ^50^ In the study, we demonstrated Smad3 signalling may be a regulator of both mitochondrial apoptosis pathway and ATF4‐CHOP apoptosis pathway. And it will inspire us to take further research to make a more extensive and intensive understanding on the crosstalk between the mitochondrial and ATF4‐CHOP apoptosis pathways in future.

In summary, our findings indicated that exogenous supplementation of APN could act as a promising therapeutic strategy for ICH injury complicated with diabetes. Furthermore, the protective effects might rely on the suppression of mitochondrial and ATF4‐CHOP apoptosis pathways via inhibition of Smad3 signalling. Our study provides a novel theoretical basis for the future clinical application of APNp in the treatment of ICH injury in diabetic patients.

## CONFLICT OF INTEREST

The authors have no conflicts of interest to declare.

## AUTHOR CONTRIBUTIONS

Y. Q. and D.‐Y. F. conducted this project and designed the study. X. W. performed the animal experiments, J.‐N. L. and H.‐X. L. performed the vitro experiments, W.‐X. C. and W. G. completed the statistics and interpreted the data. L. Z. and H. G. helped to draft the manuscript. H. B. and K. G. revised the manuscript.

## ETHICAL APPROVAL

All experimental procedures described here were approved by the Ethics Committee of the Fourth Military Medical University and were performed in accordance with the guidelines of the National Institutes of Health Guide for the Care and Use of Laboratory Animals.

## Data Availability

The data sets used and/or analysed during the current study are available from the corresponding author on reasonable request.
